# Genome-wide identification and characterization of OVATE family proteins in *Betula luminifera* reveals involvement of *BlOFP3* and *BlOFP5* genes in leaf development

**DOI:** 10.3389/fpls.2022.950936

**Published:** 2022-10-13

**Authors:** Priyanka Borah, Fei Ni, Weiyang Ying, Hebi Zhuang, Sun-Li Chong, Xian-Ge Hu, Jun Yang, Er-pei Lin, Huahong Huang

**Affiliations:** ^1^ State Key Laboratory of Subtropical Silviculture, Zhejiang A&F University, Hangzhou, China; ^2^ Shanghai Key Laboratory of Plant Functional Genomics and Resources, Shanghai Chenshan Plant Science Research Center, Chinese Academy of Sciences, Shanghai, China

**Keywords:** *Betula luminifera*, ovate family protein, expression analysis, protein-protein interaction, transmission electron microscopy

## Abstract

Ovate family proteins (OFP) are plant-specific transcription factors involved in regulating morphologies of the lateral organs, plant growth and development. However, the functional roles of *OFP* genes in *Betula luminifera*, an important timber tree species, are not well studied. In this study, we identified 20 *BlOFP* genes and analyzed their phylogenetic relationship, gene structure, conserved motifs, and *cis*-elements. Further, expression analysis indicates that *BlOFP* genes were up-regulated in leaves on the one-year-old branch compared to leaves on the current-year branch and bract, except *BlOFP7*, *BlOFP11*, *BlOFP14* and *BlOFP12*. The overexpression of *BlOFP3* and *BlOFP5* in *Arabidopsis thaliana* not only resulted in a slower growth rate but also produced sawtooth shape, flatter and darker green rosette leaves. Further investigation showed that the leaf thickness of the transgenic plants was more than double that of the wild type, which was caused by the increasement in the number and size of palisade tissue cells. Furthermore, the expression analysis also indicated that the expressions of several genes related to leaf development were significantly changed in the transgene plants. These results suggested the significant roles of *BlOFP3* and *BlOFP5* in leaf development. Moreover, protein-protein interaction studies showed that BlOFP3 interacts with BlKNAT5, and BlOFP5 interacts with BlKNAT5, BlBLH6 and BlBLH7. In conclusion, our study demonstrates that BlOFP3 and BlOFP5 were involved in leaf shape and thickness regulation by forming a complex with BlKNAT5, BlBLH6 and BlBLH7. In addition, our study serves as a guide for future functional genomic studies of *OFP* genes of the *B. luminifera*.

## Introduction

The OVATE Family Proteins (OFP) are unique plant-specific transcription factors containing a ~70 amino acids long conserved C-terminal OVATE domain (DUF623) (Schmitz et al., 2015). Based on the similarity of the OVATE domain, OFPs were identified in monocots, dicots, bryophytes and lycophytes (Liu et al., 2014). The first *OFP* gene was cloned by Liu et al. (2002) from tomatoes, and they reported that a point mutation in the *OFP* gene resulted in pear-shaped tomatoes, and overexpression causes abnormal phenotypes like slower plant growth, exserted stigmas, and altered floral and vegetative architecture. Later, the function of *OFP* genes was studied in many plant species and identified that *OFP* genes exhibit various roles in the different stages of a plant’s growth and development. For example, in *Arabidopsis thaliana*, an overexpression study of *AtOFP1* exhibits round and curly leaves and kidney-shaped cotyledons. Moreover, the expression of *AtOFP1* also inhibits the expression of *AtGA20ox1*, an essential gene for gibberellin biosynthesis, and prevents cell elongation (Wang et al., 2007). Besides, Wang et al. (2011) reported that in *A. thaliana*, *AtOFPs* act as transcriptional repressors regulating the morphology of cotyledons and true leaves, flower formation, and seed development. Further, overexpression of *OsOFP2* in rice leads to a decrease in plant height and changes in leaf morphology and seed shape (Schmitz et al., 2015). Likewise, genetic transformation experiments revealed that rice *OsOFP19* could affect architecture and seed shape by regulating the BR signalling pathway (Yang et al., 2018).

OFP proteins form complexes with other proteins and directly or indirectly affect the transcription of target genes to regulate plant growth and development. For example, *A. thaliana* OFPs can interact with TALE (three-amino-acid-loop-extension) superfamily members to form a complex and play an essential regulatory role in growth and development (Hackbusch et al., 2005). Further, AtOFP1 and AtBLH3 form a protein complex that regulates the transition time from vegetative growth to reproductive development of *A. thaliana* (Zhang et al., 2016). Moreover, AtOFP4 interacts with AtKNAT7 protein and acts as a transcriptional inhibitor to regulate the formation of the secondary cell wall of the inflorescence stem in *A. thaliana* (Li et al., 2011; Li et al., 2012). In addition, AtOFP5 interacts with AtBLH1 and AtKNAT3 and coordinately regulates embryo sac development (Pagnussat et al., 2007). Besides this, AtOFP1 also interacts with DNA double-strand break repair-related protein AtKu and jointly regulates the DNA double-strand break repair process (Wang et al., 2010). Further, rice OsOFP8 phosphorylates OsGSK2 protein and plays a role in growth and development by participating in the BR signalling pathway (Yang et al., 2016). Likewise, OsOFP19 forms a complex with OSH1 (a member of the KNOX subfamily protein) and DLT (DWARF AND LOW-TILLERING) protein. The complex of these three proteins plays a vital role in regulating BR signalling and determining cell division patterns (Yang et al., 2018).

*Betula luminifera* H. Winkler is a deciduous tree of the Betulaceae family, distributed in 14 provinces of southern China (Li et al., 2018). It grows fast and has strong adaptability with a high yield rate (Li et al., 2018). In addition to these traits, it has a short juvenile period. The one-and-a-half-year-old young plants can bloom under natural conditions, making the corresponding breeding and research cycle faster than other tree species (Li et al., 2018). Further, the complete genome sequence of the same genus, *Betula pendula* has been published (Salojarvi et al., 2017). These special characteristics make *B. luminifera* an ideal plant species for forest genetic and molecular biology studies. In recent years, bioinformatics and molecular biology studies of *B. luminifera* have also been conducted, such as RNA-seq analysis and gene family studies (Cai et al., 2018; Li et al., 2018). However, so far, there are no research findings involving the OFP transcription factor of *B. luminifera*. Therefore, in this study, we identified the *OFP* gene family in *B. luminifera* and analyzed their gene structure, conserved domains, evolutionary relationships, and expression patterns in different developmental stages of leaves. Moreover, functional studies for *BlOFP3* and *BlOFP5* were performed through ectopic expression in *A. thaliana* and protein-protein interactions. The present results provide a basis for functional studies of *OFP* genes in *B. luminifera*.

## Materials and methods

### Plant materials

The material used in this study is *B. luminifera* 1-V-25-2 clone, planted in the Pingshan nursery of Zhejiang A & F University. The leaves of three different developmental stages were collected from a healthy plant and named Brt (bracts), YL1 (young leaf from the current-year branch), and YL2 (young leaf from the one-year-old branch). The sample was collected in April 2021. The *A. thaliana* Columbia-0 (Col-0) was used for gene transformation. Seeds of Col-0 were sterilized twice with 75% alcohol and grown on ½ MS media until leaves reached the four-leaf stage in an artificial incubator (at 25°C, light for 16 h, dark for 8 h, with an average light intensity of 12,000 lx). Then the seedlings were transplanted to the peat moss (peat soil: vermiculite: perlite is 3:1:1) and grew at 22°C under photoperiod (16h light/8h dark) in a growth chamber.

### Identification of *OFP* genes

The Hidden Markov Model (HMM) profile of the OVATE domain (PF04844) was obtained from the Pfam database (Mistry et al., 2021). Putative OFP protein sequences were identified by HMMER search against the *B. luminifera* genome and the publicly available *B. pendula* genome (Salojarvi et al., 2017). In cases of multiple putative proteins from the same gene locus, the most extended variant was kept for further analysis. Editseq module in the DNAStar package was used to predict the amino acid sequence, molecular weight, and isoelectric point of the deduced protein. The identified gene sequences are provided in **Supplementary Table 1**.

### Gene structure, genome location and *cis*-elements analysis of *BlOFPs*

The gene structure and genomic location of *BlOFPs* were searched in the genomic database, and the localization of the *BlOFP* genes on the chromosomes was generated using TBtools (Chen et al., 2020). The PlantCARE webtool (http://bioinformatics.psb.ugent.be/webtools/plantcare/html/) was used to analyze the *cis-*acting elements in the promoter sequence.

### Phylogenetic analysis and conserved motif analysis of BlOFP proteins

The phylogenetic tree was constructed using MEGA-X using the Maximum likelihood method (Kumar et al., 2018). The aligned peptide sequences of OVATE domains from *B. luminifera*, *B. pendula*, *A. thaliana*, *Oryza sativa* and *P. trichocarpa* OFP proteins were used to generate phylogenetic analysis. The bootstrap value of 1000 replicates was used to evaluate the statistical significance. The conserved motifs were identified by the online program MEME (http://meme-suite.org/tools/meme) and visualized using the TBtools (Chen et al., 2020).

### RNA isolation

According to the manufacturer’s instructions, the total RNA was extracted from tissues using PureLinkTM Plant RNA Reagent (Invitrogen, Carlsbad, CA, USA). The concentration of RNA samples was analyzed by spectrophotometer (Nanodrop ND-2000), and purity and integrity were analyzed by agarose gel electrophoresis. Promega’s RQ1 RNase-Free DNase was used to remove the DNA contamination following the manufacturer’s instructions.

### Real-time qRT-PCR

The cDNA was synthesized using Takara’s PrimeScript™ RT reagent Kit (Perfect Real Time) as per the manufacturer’s instructions. Oligo7.0 software was used to design fluorescent quantitative PCR primers (**Supplementary Table 2**). The TB green premix ex Taq II (Tli RNase H Plus) (Takara) was used for qRT-PCR amplification in the CFX96™ Real-Time PCR detection system (Bio-Rad, USA). All PCR reactions were performed in triplicate. The *BlEF1a* gene was used as an internal reference for *B. luminifera* samples, and the *Actin* gene was used as an internal reference in qRT-PCR for *Arabidopsis* samples. All the primers used in qRT-PCR were listed in **Supplementary Table 2**. The relative expression levels were determined with CFX96 Manager™ software (version 1.6; Bio-Rad, USA) using the delta-delta Ct method (Livak and Schmittgen, 2001). GraphPad Prism (Version 5.01) was used to draw the graphs of the quantitative results.

### Production of transgenic *A. thaliana* plants

For the construction of the overexpression vector, the open reading frame (ORF) of *BlOFP3* and *BlOFP5* genes were inserted into the pCAMBIA13011 vector, respectively. The generated vectors *pCAMBIA13011-BlOFP3* and *pCAMBIA13011-BlOFP5* were transferred to *Agrobacterium tumefaciens* (GV3101). Transformation of transgenic *A. thaliana* plants was performed using the floral dip method (Clough and Bent, 1998), and transgenic plant selection was performed on MS agar medium supplemented with hygromycin. The transgenic plants were transplanted to peat moss and grown as mentioned above for wild-type (WT) plants. The transgenic *A. thaliana* plants were also verified by PCR and qRT-PCR, and homozygous T3 generation plants were used for further analysis.

### Phenotyping of transgenic plants

To observe the growth and leaf development of transgenic plants, the young (21-day-old) and mature (42-day-old) transgenic plants were photographed and compared with WT. The relative chlorophyll content of leaves was measured using a SPAD (soil plant analysis development) meter. To measure the leaf thickness and cell number, the cross-section of about 8 μm thick from rosette leaves of 42-day-old plants was obtained by microtome (Leica). The sections were stained with 0.02% toluidine blue for 5 minutes and rinsed with distilled water until the wash solution was clear. Finally, the sections were observed under a light microscope (Zeiss). At least 8 sections (8 replicates) of each sample were used to determine the leaf thickness and cell number.

### Transmission electron microscopy

To measure cell size, the rosette leaves of a 42-day-old plant were fixed in 0.1 M phosphate buffer (pH 7.2) with 2.5% glutaraldehyde at 4°C overnight. Then, the samples were washed with 0.1 M phosphate buffer and treated with 1.0% osmium tetroxide for 1h. After that, the samples were rinsed twice in deionized water and dehydrated in a graded ethanol series. After dehydration, the samples were infiltrated and embedded in epon-araldite. Ultra-thin sections (70 nm) were sliced with a diamond knife (Reichert-Jung, Germany), stained with uranyl acetate and lead citrate, and mounted on nickel grids for examination in the transmission electron microscope (FEI Titan G2 80-200 ChemiSTEM, USA). At least 10 photos for each sample were analyzed.

### Yeast two-hybrid assay

Y2H experiment was performed according to the manufacturer’s instructions of Matchmaker™ gold yeast two-hybrid system (Clontech). Briefly, the ORFs of *BlOFPs* were cloned into bait vector pGBKT7, respectively. The ORFs of *BlKNOXs* and *BlBLHs* genes were constructed into prey vector pGADT7, respectively. The recombinant bait and prey vectors were transformed into the Y2H gold strain and Y187 strain, and the protein interaction was tested through yeast mating according to the manufacturer’s instructions. The pGADT7-T+pGBKT7-Lam and pGADT7-T+pGBKT7-53 were used as negative and positive controls, respectively. The gene sequences used in Y2H were provided in **Supplementary Table 1**, and the primers used in Y2H were also listed in **Supplementary Table 2**.

### Bimolecular fluorescence complementation assay

The ORF of BlOFP3, BlOFP5, BlKNOX5, BlKNOX9, BlBLH6, BlKNOX5, BlKNOX9 and BlBLH7 were cloned into the pSAT1- nEYFP-C1 and pSAT4-cEYFP-C1-B vectors using ClonExpress^®^ II One Step Cloning Kit (Vazyme). The recombinant vectors transiently co-expressed in *Arabidopsis* protoplast cells through PEG-mediated transformation (Yoo et al., 2007). The images of transformed protoplast cells were captured on a Laser confocal microscope (Carl Zeiss LSM880) to observe and detect protein interaction signals. The primers used in BiFC were listed in **Supplementary Table 2**.

### Statistical analysis

The significant differences in experimental data were compared by the Duncan algorithm. All calculations are based on Data Processing System Software (V15.10), and differences were considered significant at a p-value ≤ 0.05 level.

## Results

### Identification of *OFP* genes in *B. luminifera* and *B. pendula*

**Figure 1:**
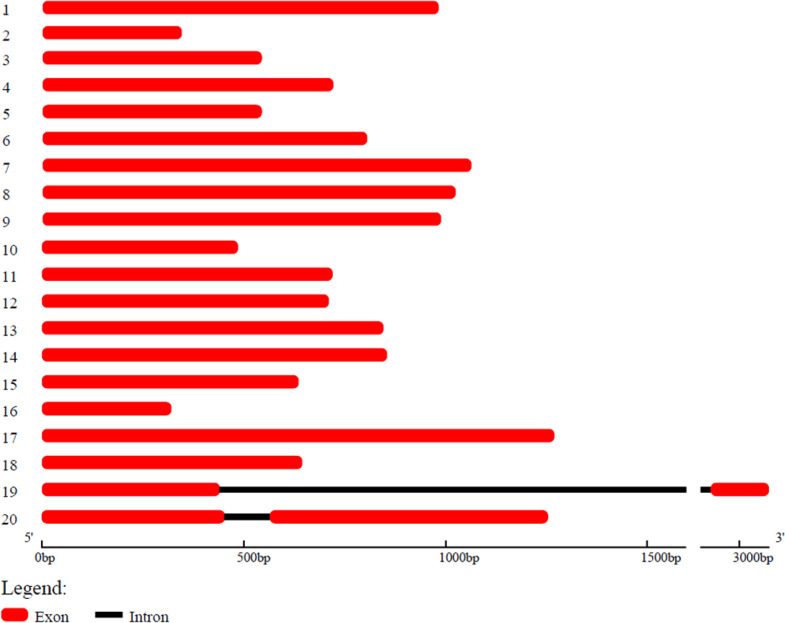
Gene structure analysis of *BlOFP* genes. The scale bar is shown at the bottom.

**Figure 2:**
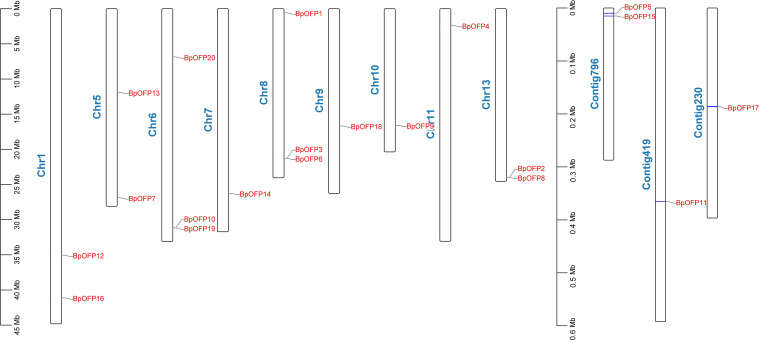
Physical location of *BpOFP* genes on the Chromosomes and scaffolds of *B. pendula*.

Our lab has developed *B. luminifera* genome reads using PacBio’s RS II platform, and the assembled genome contained 9,332 contigs with an N50 of 247 kb (unpublished data). Using this contigs information, we have identified a total of 20 *B. luminifera BlOFPs* (*BlOFP1*-*B1OFP20*) genes using the HMM model. Further, we also identified the 20 *BpOFPs* of silver birch (*B. pendula*) using the published genome (Salojarvi et al., 2017). The 20 B1OFP proteins consist of 106-422 amino acid residues, with a molecular weight of 12.08-48.21 kDa and an isoelectric point of 4.38-9.79 (**Table 1**). Further, multiple sequence alignment indicated that all the 20 BlOFP proteins contain an OVATE domain, which was not highly conserved among these proteins (**Supplementary Figure 1**). The gene structure of *BlOFP* genes is also analyzed and identified that the ORF size in the genomic DNA ranged from 321 - 1269 bp. Out of 20 *BlOFP* genes, 18 *BlOFPs* genes are intron-free. While *BlOFP19* and *BlOFP20* have an intron of 2506 bp and 110 bp in length, respectively (**Figure 1**). Based on the published genome database of *B. pendula* (Salojarvi et al., 2017), we analyzed the distribution of *BpOFPs* genes on the chromosomes (**Figure 2**). The 16 *BpOFPs* genes are mapped to 9 chromosomes of *B. pendula*, of which chromosomes 6 and 8 have the highest number of genes, i.e. three *OFPs*. In addition, *BpOFP5*, *BpOFP11*, *BpOFP15*, and *BpOFP17* were found to be located on unanchored contig796, contig419 and contig230 (**Figure 2**).

**Table 1:** Physical and chemical properties of *BlOFP* genes in *B.luminifera*.

S. No	cDNA	ORF size (bp)	Predicted protein length/m.w.(kDa)	Isoelectric point	S. No	cDNA	ORF size (bp)	Predicted protein Length/m.w.(kDa)	Isoelectric point
1	BlOFP1	981	326/36.60	9.79	1	BpOFP1	981	326/36.59	9.94
2	BlOFP2	345	114/12.92	4.53	2	BpOFP2	345	114/12.92	4.53
3	BlOFP3	543	180/20.67	8.7	3	BpOFP3	543	180/20.70	8.7
4	BlOFP4	720	239/26.47	4.38	4	BpOFP4	720	239/26.58	4.29
5	BlOFP5	543	180/20.24	5.11	5	BpOFP5	543	180/20.14	4.87
6	BlOFP6	804	267/29.53	4.5	6	BpOFP6	729	242/26.70	4.3
7	BlOFP7	1062	353/39.98	9.6	7	BpOFP7	1062	353/40.10	9.54
8	BlOFP8	1023	340/38.03	9.38	8	BpOFP8	1020	339/38.00	9.33
9	BlOFP9	987	328/38.21	9.71	9	BpOFP9	894	297/34.64	9.64
10	BlOFP10	486	161/18.68	7.9	10	BpOFP10	474	157/18.21	8.74
11	BlOFP11	720	239/26.42	5.63	11	BpOFP11	720	239/26.46	5.96
12	BlOFP12	711	236/26.69	6.97	12	BpOFP12	711	236/26.67	6.59
13	BlOFP13	846	281/31.58	6.12	13	BpOFP13	846	281/31.69	6.62
14	BlOFP14	855	284/32.78	8.15	14	BpOFP14	777	258/29.74	9.35
15	BlOFP15	636	211/24.15	8.86	15	BpOFP15	642	213/24.21	8.9
16	BlOFP16	321	106/12.08	4.54	16	BpOFP16	324	107/12.26	4.54
17	BlOFP17	1269	422/48.21	9.21	17	BpOFP17	1197	398/45.57	9.4
18	BlOFP18	645	214/24.35	6.07	18	BpOFP18	645	214/24.16	5.23
19	BlOFP19	585	194/23.02	8.54	19	BpOFP19	561	186/21.64	7.69
20	BlOFP20	1143	381/43.12	9.36	20	BpOFP20	330	110/12.82	10.29

### Phylogenetic analysis of birch OFP proteins

**Figure 3:**
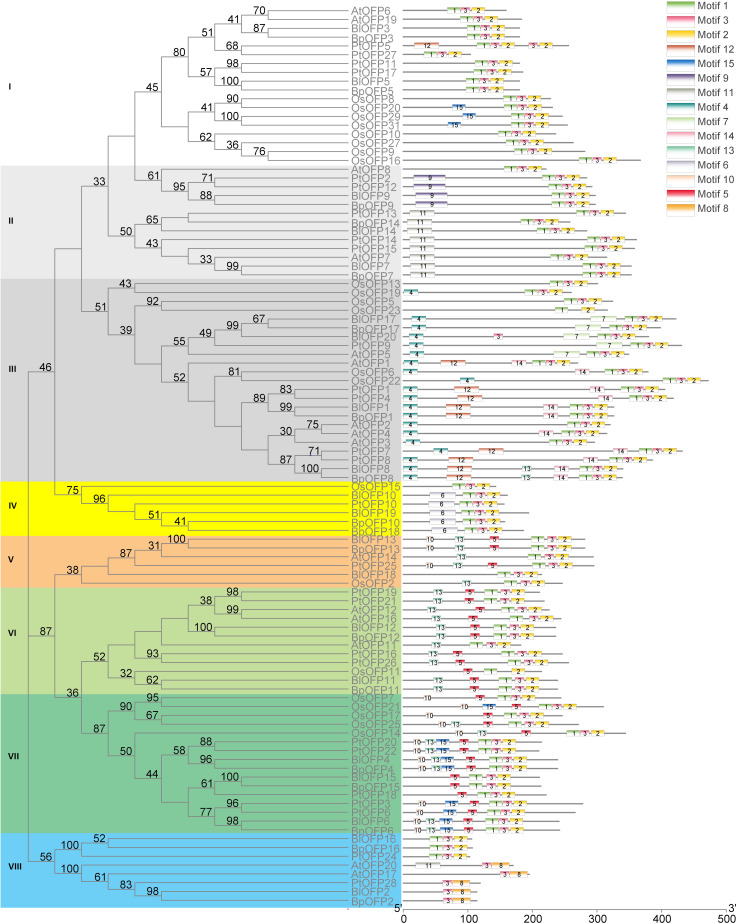
Phylogenetic relationship and conserved motif analysis of BlOFPs from *B. luminifera*. The phylogenetic tree was constructed using aligned OVATE domains sequences of *B. luminifera* (Bl), *B. pendula* (Bp), *A. thaliana* (At), *Oryza sativa* (Os) and *P. trichocarpa* (Pt). All the eight subgroups are indicated in different colours. The conserved motifs (1-15) are indicated in 15 different colour boxes.

In order to analyze the phylogenetic relationship of OFP proteins, a phylogenetic tree containing 102 OFP proteins (BlOFPs, BpOFPs, AtOFPs, OsOFPs and PtOFPs) is constructed. All OFP proteins can be divided into eight sub-groups from the topological structure: Subgroup I, Subgroup II, Subgroup III, Subgroup IV, Subgroup V, Subgroup VI, Subgroup VII and Subgroup VIII (**Figure 3**). Except for BlOFP18 and BpOFP18, other BlOFP and BpOFP proteins are placed next to each other in all clades, indicating that these OFP proteins have a certain degree of differentiation during evolution. The two concerned BlOFPs of the present study, i.e., BlOFP3 and BlOFP5, were placed in the same subgroup, Subgroup-I. Along with BlOFP3 and BlOFP5, AtOFP6 and AtOFP19 from *A. thaliana* and OsOFP8 and OsOFP20 from *O. sativa* are grouped in the same subgroup, indicating their close homologous relationships (Liu et al., 2014). Further, most monocot OFP proteins (OsOFPs) are divided into six subgroups, except Subgroup II and VIII.

### Prediction of conserved motifs in birch OFP proteins

Studying the conserved motifs can reveal the functional diversity of OFP proteins. Through conserved motif analysis, fifteen conserved motifs of BlOFPs were identified. Among these motifs, OVATE domains are present in the C-terminal of OFP proteins, while other motifs are upstream of the OVATE domain (**Figure 3**). Interestingly, the OsOFP23 in Subgroup III, OsOFP11 in Subgroup VI and BlOFP2, BpOFP2, PtOFP28, AtOFP17 and AtOFP20 in the Subgroup VIII don’t contain the complete OVATE domain (**Figure 3**). These results suggest that the OVATE domain is not that conserved during evolution. Further, most OFP proteins in the same subgroup have similar motif distributions (**Figure 3**). These results show that the motifs of members within the same subgroup are conserved, which implies they may perform similar functions.

### Analysis of *cis-*acting elements in the promoter region of *BlOFP* genes

**Figure 4:**
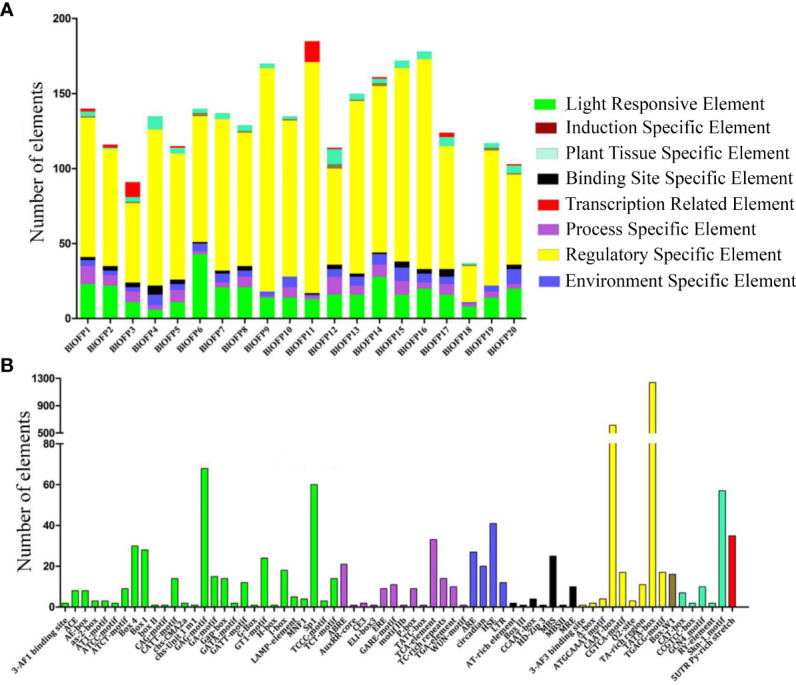
Analysis of conserved *cis*-acting elements in the promoter region of BlOFPs. Elements were identified in the 1500 bp sequences upstream of the start codon of *BlOFP* genes. **(A)** The elements involved in specific functions are indicated by different colours for each gene. **(B)** Number of different conserved elements.

The *cis-*acting elements play a vital role in regulating gene transcription during plant growth, development, and stress. Therefore, studying the *cis*-elements in *BlOFP* genes will help understand their role in plant growth and development. Hence, we analyzed the 1500 bp upstream region of the 20 *BlOFPs* genes. We classified the *cis-*acting elements into eight types based on the functions of *cis-*elements: light-responsive elements, induction-specific elements, plant tissue-specific elements, binding site-specific elements, transcription-related elements, process-specific elements, regulatory-specific elements, and environment-specific elements (**Figure 4A**). The promoter regions of *BlOFPs* genes contain at least six different types of *cis-*acting elements. Although the regulatory-specific *cis-*elements are the most abundant among the different *cis-*elements analyzed, the process-specific elements (plant hormone-related *cis*-acting elements) seem to be essential. Further, ABRE, ERE, GARE motif, TCA-element, TC-rich repeats, and TGA elements were found in abundance compared to other process-specific elements (**Figure 4B**).

### Expression of *BlOFP* genes during leaf development in *B. luminifera*

**Figure 5:**
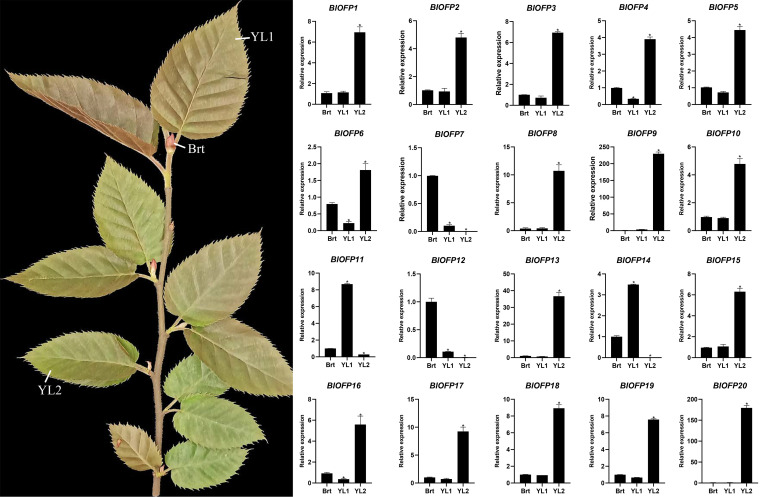
Expression analysis of *BlOFP* genes for leaf development. qRT-PCR analysis of 20 *BlOFP* genes in three developmental stages of *B. luminifera* leaves. The error bar represents the standard deviation from three biological replicates. The asterisk indicates significant difference compared to Brt at *p*<0.05 level.

The *OFP* genes are reported to be involved in the regulation of leaf development (Yang et al., 2016; Sun et al., 2020). So to identify the expression pattern of *BlOFP* genes in leaf development, we performed qRT-PCR in *B. luminifera* leaves with three developmental stages: Brt, YL1, and YL2. Most *BlOFP* genes have increased expression in YL2 except *BlOFP7*, *BlOFP11*, *BlOFP14*, and *BlOFP12* (**Figure 5**). The *BlOFP7* and *BlOFP12* showed their highest relative expressions in Brt, and the *BlOFP11* and *BlOFP14* showed their highest expressions in YL1 (**Figure 5**). Interestingly, our qRT-PCR analysis showed that the *BlOFP9* and *BlOFP20* gene expression in YL2 was 228 and 184 times to the Brt, and 63 and 162 times to YL1, respectively. Further, *BlOFP3* and *BlOFP5* in YL2 were 6.9 and 4.4 times to the Brt and 9.6 and 6.2 times to the YL1, respectively (**Figure 5**).

### Phenotype and anatomic analysis of *Arabidopsis* overexpressing *BlOFP3* and *BlOFP5*

**Figure 6:**
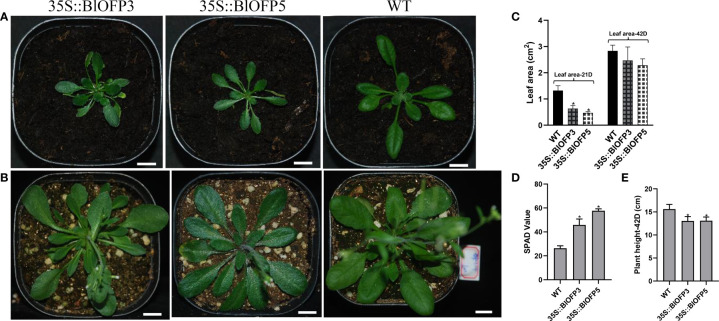
Phenotype observation on transgenic plants overexpressing *BlOFP3* and *BlOFP5* in *A. thaliana*. **(A)** 21-day-old transgenic plants transformed with 35S::BlOFP3 (left), 35S::BlOFP5 (middle), and WT plants (right). **(B)** 42-day-old transgenic plants transformed with 35S::BlOFP3 (left), 35S::BlOFP5 (middle), and WT plants (right). **(C)** Leaf area of 21-day and 42-day-old plants. **(D)** SPAD values of plants. **(E)** Plant height of 42-day-old plants. Bar = 1cm. The asterisk indicates significant difference compared to WT at *p*<0.05 level.

In our study, through phylogenetic and motif analysis, we identified that *AtOFP6* and *OsOFP8* are closely related to *BlOFP3* and *BlOFP5*. Furthermore, sequence alignment of AtOFP6, BlOFP3 and BlOFP5 showed that conserved OVATE domain among these sequences (**Supplementary Figure 2**). Considering the function of *AtOFP6* in leaf development (Wang et al., 2011), the role of *BlOFP3* and *BlOFP5* genes was first investigated through ectopic expression in *A. thaliana* plants. The transgenic plants were screened out and confirmed using PCR detection on two reporter genes and BlOFP3/5 (**Supplementary Figure 3**), and then homologous T3 generations were used for phenotypic observation and anatomical analysis. The expression of *BlOFP3* and *BlOFP5* genes are also investigated by qRT-PCR in T3 generation transgenic plants. The results showed that *BlOFP3* and *BlOFP5* were strongly expressed in the transgenic plants (**Supplementary Figure 3**). Phenotype observation showed that overexpressing of *BlOFP3* and *BlOFP5* caused apparent differences in growth rate and leaf shape (**Figure 6**). The plant height of 42-day-old transgenic plants was 13.04 cm (35S::*BlOFP3*) and 13.08 cm (35S::*BlOFP5*), respectively, while the plant height of WT was 15.60 cm, which indicated that the growth of transgenic plants was slower (**Figure 6E**). Moreover, the rosette leaves of the two transgenic lines were flatter, darker green in colour, and a more obvious sawtooth appeared near the leaf stalk (**Figures 6A, B**). Measurement of the leaf area of 21-day-old and 42-day-old plants showed that the plants overexpressing *BlOFP3* and *BlOFP5* have considerably less leaf area (**Figure 6C**).

**Figure 7:**
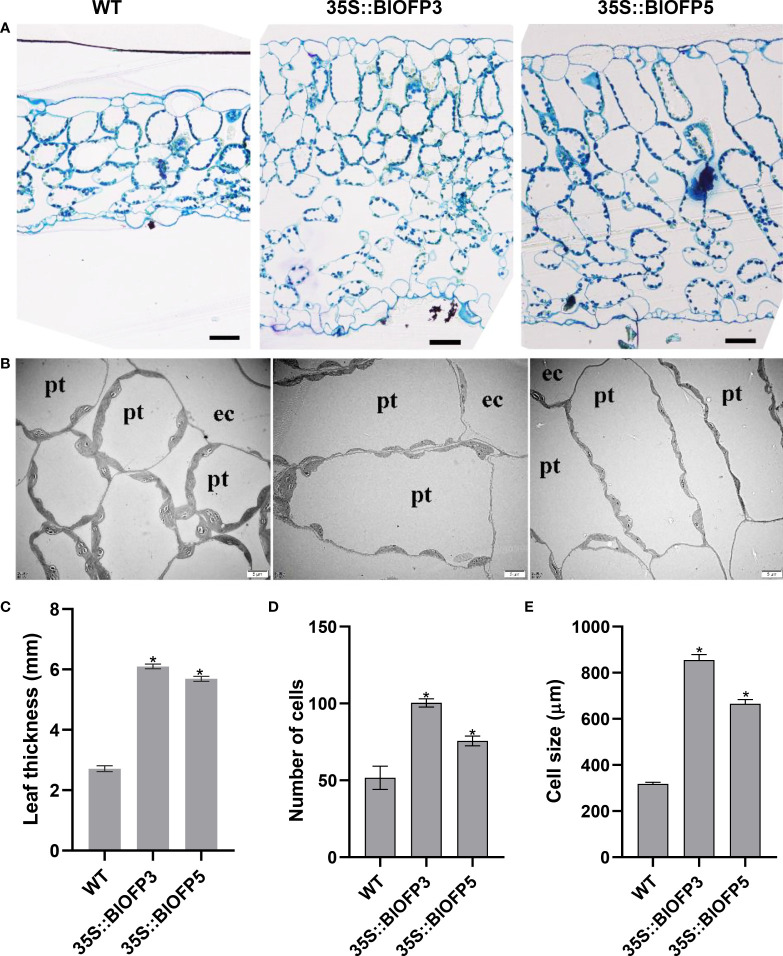
Anatomical characterization of transgenic Arabidopsis leaves. **(A)** Transverse sections of leaves from WT and transgenic *A. thaliana* plants stained with toluidine blue. Bar = 50 µm. **(B)** The TEM analysis of transgenic *A. thaliana* leaves. Bar = 5 µm. **(C)** Leaf thickness of WT and transgenic plants. **(D)** Number of palisade tissue cells. **(E)** Size of palisade tissue cells The asterisk indicates significant difference compared to WT at *p*<0.05 level.

Furthermore, the SPAD values indicated that the relative chlorophyll content of leaves in transgenic plants is significantly higher than that in WT (**Figure 6D**). And the anatomic analysis revealed that the leaf thickness of plants overexpressing *BlOFP3* and *BlOFP5* is more than two times of the WT (**Figures 7A, C**). Meanwhile, an obvious increase in the number and size of palisade tissue cells is also observed in transgenic plants (**Figures 7A, D, E**). The increase in the size of palisade tissue cells was confirmed using transmission electron microscopy (TEM) (**Figure 7B**). Besides, the expression of *AtOFP6*, the endogenous homolog for *BlOFP3* and *BlOFP5*, was not altered in the plants overexpressing *BlOFP3* and *BlOFP5* (**Supplementary Figure 4**). These results suggest that *BlOFP3* and *BlOFP5* may play essential roles in leaf development by regulating the cell’s number and size in palisade tissue.

### Expression analysis of genes associated with leaf development in transgenic *Arabidopsis*

**Figure 8:**
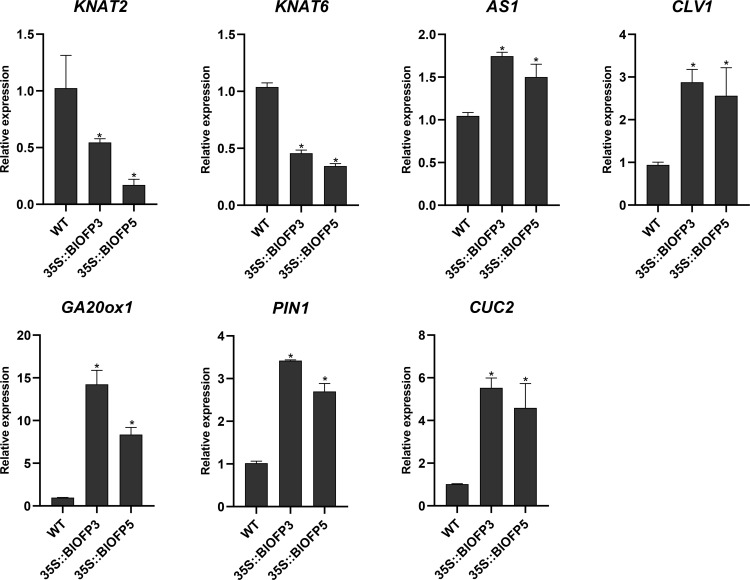
Expression analysis of genes involved in leaf development. qRT-PCR analysis of *KNAT2*, *KNAT6*, *AS1*, *CLV1*, *GA20ox1*, *PIN1* and *CUC2* genes normalized with *Actin*. The error bar indicates the standard deviation from three biological replicates. The asterisk indicates significant difference compared to WT at *p*<0.05 level.

To explore the molecular mechanism of *BlOFP3* and *BlOFP5* in leaf development, six genes, including *KNAT2*, *KNAT6*, *AS1*, *CLV1*, *GA20ox1*, *PIN1* and *CUC2* genes, have been proved as crucial genes in leaf development (Long et al., 1996; Belles-Boix et al., 2006; Wang et al., 2021; Zhou et al., 2022), were chosen for expression analysis in the transgenic *A. thaliana* plants. As shown in figure 8, the expressions of *KNAT2* and *KNAT6* were both drastically inhibited in plants overexpressing *BlOFP3* and *BlOFP5* (**Figure 8**). At the same time, the expression of *CLV1*, *GA20ox1*, *PIN1* and *CUC2* genes up-regulated in the transgenic plants (**Figure 8**). These results further support the essential roles of *BlOFP3* and *BlOFP5* in leaf development. It also implies that these two genes may perform redundant functions through similar pathways in the leaf development of *B.luminifera*.

### Interaction analysis of BlOFP3 and BlOFP5 with TALE homeodomain protein

**Figure 9:**
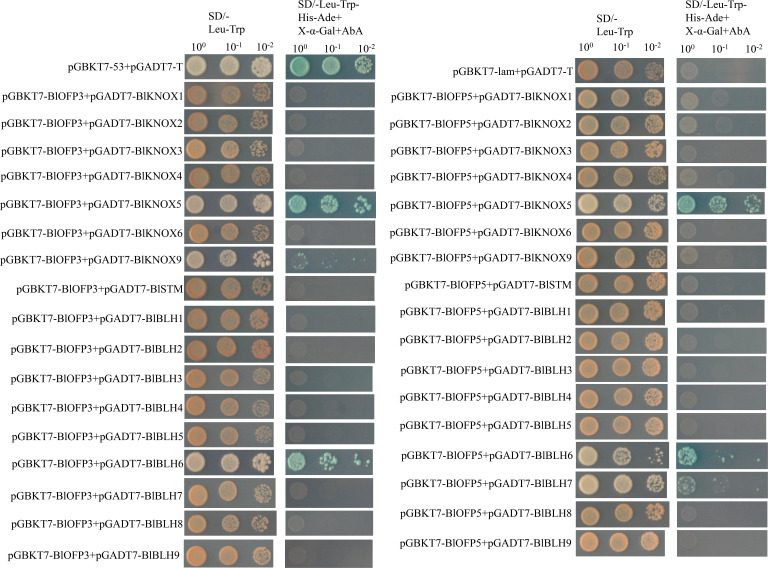
Protein-protein interactions of BlOFP3 and BlOFP5 with eight BlKNOX proteins and nine BlBLH proteins tested by Y2H. Yeast cells co-transformed with pGBKT7-53 and pGADT7-T were used as the positive control, and pGBKT7-lam and pGADT7-T were used as a negative control. Positive interactions were specified by the ability of cells to grow on SD-Leu-Trp-His-Ade plates supplemented with 40 μg/ml X-α-Gal and 125 ng/ml Aureobasidin A. Images were taken after 5 days of incubation at 30°C.

In previous studies, OFP proteins have been shown to interact with various TALE (KNOX and BLH) proteins, forming a TALE-OFP protein complex to regulate different biological processes (Hackbusch et al., 2005; Li et al., 2011; Liu and Douglas, 2015). In this study, the interactions between these two BlOFP proteins (BlOFP3 and BlOFP5), eight BlKNOX proteins and nine BlBLH proteins were first analyzed using the Y2H method, respectively. As shown in figure 9, strong interactions between BlOFP3 and BlKNOX5, BlOFP3 and BlBLH6, BlOFP5 and BlKNOX5 as well as BlOFP5 and BlBLH6 were detected, and weak interactions between BlOFP3 and BlKNOX9, and BlOFP5 and BlBLH7 were also identified. However, in the present experiment, all other members of TALE homeodomain proteins did not show any interactions with BlOFP3 and BlOFP5 (**Figure 9**). This means there is interaction specificity exists between OFP and TALE homeodomain proteins.

**Figure 10:**
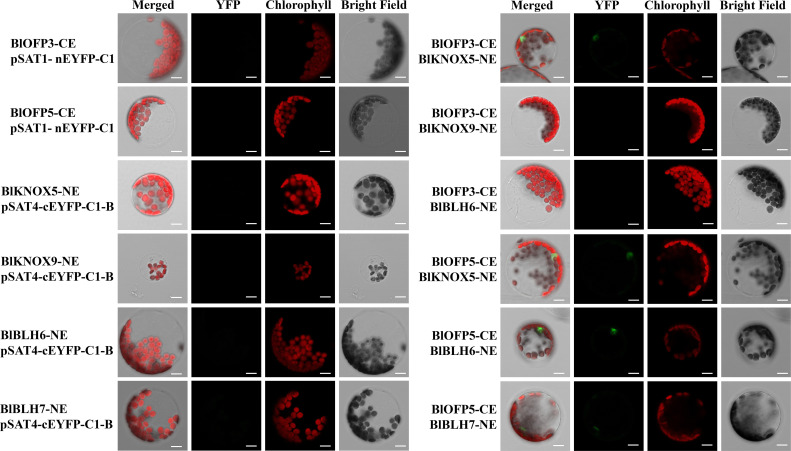
Verification of positive interactions of BlOFP3 and BlOFP5 with TALE proteins by BiFC assay in protoplast cells of *A. thaliana*. Proteins fused to the N- or C-terminal fragments of EYFP (NE or CE, respectively) were co-expressed transiently in protoplasts. Fluorescence signal indicating reconstitution of YFP was analyzed by confocal laser scanning microscopy. Empty vectors and corresponding recombinant vectors were co-expressed as negative control. Bar = 10 μm.

Furthermore, the interactions obtained from Y2H were then verified by the BiFC assay in mesophyll protoplast cells of *A. thaliana*. Similar to the Y2H assay, the protoplast cells co-expressing BlOFP3-cEYFP with BlKNOX5-nEYFP, BlOFP5-cEYFP with BlKNOX5-nEYFP, as well as BlOFP5-cEYFP with BlBLH6-nEYFP exhibited intense fluorescence signals (**Figure 10**). In addition, a weak fluorescence signal was detected in the protoplast cells co-expressing BlOFP5-cEYFP with BlBLH7-nEYFP (**Figure 10**). These results were consistent with the Y2H interactions. However, in contrast to Y2H results, the fluorescence signal was absent in protoplast cells co-expressing BlOFP3-cEYFP with BlKNOX9-nEYFP and BlOFP3-cEYFP with BlBLH6-nEYFP (**Figure 10**). Based on the above results, we hypothesized that at least BlKNOX5 is a common partner for BlOFP3 and BlOFP5, forming a functional complex to regulate leaf development in *B.luminifera*.

## Discussion

OFPs, encoding a class of plant-specific transcription regulators, have been identified in many plants (Wang et al., 2010; Rodríguez et al., 2011; Tsaballa et al., 2011; Wang et al., 2011; Wang et al., 2016; Yang et al., 2016; Yang et al., 2018 **).** This study identified and characterized 20 genes encoding OFP proteins in *B. luminifera*. As indicated by phylogenetic analysis, 20 OFP proteins in *B. luminifera* are distributed into eight subgroups based on their sequence similarity (**Figure 3**). The two studied BlOFPs of the present study, i.e., BlOFP3 and BlOFP5, showed close homologous relationships with AtOFP6 and AtOFP19 from *A. thaliana*. Domains and motifs are the key elements for the structure and functions of a protein or protein family (Moore et al., 2008; Forslund and Sonnhammer, 2012). In this study, 15 conserved motifs were identified, and most of these motifs showed similar distribution patterns to their homologs within the same subgroup (**Figure 3**). Moreover, the OVATE domain was also identified to be present at the end of 20 BlOFPs (**Figure 3**), which is consistent with the previous studies (Liu et al., 2014; Li et al., 2019). Since BlOFPs and BpOFPs share almost identical OVATE domain sequences, all BlOFPs and BpOFPs are positioned next to each other except BlOFP18 and BpOFP18. Divergent expansion might be considered the reason behind the presence of BlOFP18 and BpOFP18 in different subgroups (Liu et al., 2014). In the current study, hormone-specific *cis*-acting elements, including ABRE, TCA-element, TC-rich repeats, and TGA elements, which are associated with abscisic acid (ABA), salicylic acid (SA), gibberellin (GA) and auxin (IAA) responses, respectively, were found to be distributed in the promoter region of the *BlOFPs* genes (**Figure 4**). These hormone-responsive regulatory elements in the promoter regions suggest that *BlOFP* genes might be involved in response to various plant hormones and developmental processes (Liu and Douglas, 2015; Tang et al., 2018; Wu et al., 2018).

*OFPs* play diverse roles at different stages of plant growth and development (Wang et al., 2016; Knaap and Ostergaard, 2018). Regulation of leaf development is one of those important functions for plant *OFP* genes. For example, Wang et al. (2011) reported that overexpression of the *AtOFP6* gene resulted in flat, thick and cyan rosette leaves in *A. thaliana*. In the current study, transgenic *A. thaliana* overexpressing *BlOFP3* and *BlOFP5* genes showed flat, thicker leaves with higher relative chlorophyll content (**Figure 6**). Likewise, Zhou et al. (2019) found that tomato plants overexpressing *SlOFP20* genes showed shorter compound leaves and contained more chlorophyll than wild plants. These observations indicate that these *OFP* genes, including *BlOFP3* and *BlOFP5*, are essential regulators for leaf development. Moreover, in transgenic *Arabidopsis* plants overexpressing *BlOFP3* and *BlOFP5*, the expressions of several essential leaf development genes (*KNAT2*, *KNAT6*, *AS1*, *CLV1*, *GA20ox1*, *PIN1*, and *CUC2*) were considerably changed (**Figure 8**). As a critical TF in plant growth and development, the class I *KNOX* genes (*KNAT2*, *KNAT6*) maintain the function of shoot apical meristem (SAM) by regulating other key genes involved in leaf development. Prior studies have reported that AS1 and AS2 form a protein complex that inhibits the expression of *KNAT2*, resulting in regular leaf morphology (Li et al., 2016; Li et al., 2017). In the current study, the expression analysis of *KNAT2* and *KNAT6* in transgenic plants has shown an antagonistic relationship with *AS1* expression (**Figure 8**). There is an antagonism that exists between auxin and KNOX too. At the same time, auxins are transported by *PIN1*. The interaction among AS1, auxin and KNOX1 may directly affect the process of leaf initiation as well as leaf morphology (Hay et al., 2006). Likewise, the *GA20ox1* is involved in forming the boundary between SAM and the primary leaf primordia, which results from inhibition of the synthesis of GA by KNOX (Rosin et al., 2003; Hay and Tsiantis, 2009; Nakayama et al., 2014). Our study identified that the *GA20ox1* expression is also significantly changed in transgenic lines. At the same time, *CLV1* promotes stem cell activity in the SAM. Similarly, *CUC2* genes are also involved in leaf primordia initiation and affect the leaves’ morphology (Hasson et al., 2011). In the current study, *CUC2* expression is also found to be up-regulated in the transgenic lines (**Figure 8**). These results further demonstrated that *BlOFP3* and *BlOFP5* probably regulate leaf development through various molecular pathways.

The TALE homeodomain proteins are fundamental regulators of plant leaf development and meristem function (Blanco et al., 2009). These homeodomain TFs is conserved in animals, plants, and fungi. In plants, TALE proteins are comprised of two classes, KNOTTED1-LIKE homeobox (KNOX) and BEL1-like homeobox (BELL), which are involved in the determination and morphological development of leaves, stems, and inflorescences (Kumar et al., 2007; Hamant and Pautot, 2010; Hay and Tsiantis, 2010). The KNOX/BELL homo- and hetero- dimerization play an essential role in general TALE protein function (Hackbusch et al., 2005). Further, Hackbusch et al. (2005) also demonstrated that KNOX and BELL proteins interact with nine AtOFP proteins, regulating plant meristem and leaf development. Many other studies also showed that OFP proteins could interact with KNOX and BLH to regulate plant growth and development (Pagnussat et al., 2007; Li et al., 2011). Therefore, it is well-established that OFPs could act as the central point to interact with multiple proteins to form different regulatory complex to accurately and efficiently regulate plant growth and development. In our study, the physical interaction between BlOFP3 and BlKNOX5 as well as BlOFP5 and BlKNOX5 was detected by Y2H and BiFC experiments (**Figures 9**, **10**). This implies that BlKNOX5 should be a significant component for the complex formed by BlOFP3 or BlOFP5. Besides, several other TALE proteins, including BlKNOX5, BlKNOX9, BlBLH6 and BlBLH7 proteins, were identified to interact with BlOFP3 or BlOFP5 by either Y2H or BiFC (**Figures 9**, **10**). Although no consistent results were obtained from Y2H and BiFC, it is possible that BlOFP3 or BlOFP5 could interact with these TALE proteins to form various complexes, which may be associated with different biological processes in *B. luminifera*, but further experiments are needed to analyze the underlying molecular mechanism.

## Conclusion

In the current study, we identified 20 *BlOFP* genes and analyzed the phylogenetic analysis, conserved motifs and *cis*-elements. The qRT-PCR analysis reveals that most *BlOFP* genes were up-regulated in YL2 compared to YL1 and bract. The overexpression of *BlOFP3* and *BlOFP5* genes in *A. thaliana* produces sawtooth shape, flatter, darker green rosette leaves, indicating their involvement in leaf development. Moreover, TEM results showing a significant increase in the number and size of palisade tissue cells in transgenic plants, which resulted in an increasement in leaf thickness. Besides, expression analysis also indicated that the expressions of several genes related to leaf development were considerably changed in transgenic plants. Furthermore, physical interactions between BlOFP3, BlOFP5 and several TALE proteins were also identified. On the basis of the interaction assay, we hypothesized that at least BlKNOX5 is a common interacting protein for BlOFP3 and BlOFP5, forming a functional complex to regulate leaf development in *B. luminifera*.

## Data availability statement

The datasets presented in this study can be found in online repositories. The names of the repository/repositories and accession number(s) can be found in the article/**Supplementary Material**.

## Author contributions

EL and HH designed the research. PB, FN, WY and HZ performed the experiments. JY performed the BiFC assay and revised the manuscript. PB, FN, WY, HZ, SC and XH analyzed the data. PB, EL and HH wrote the manuscript with comments from all authors. All authors contributed to the article and approved the submitted version.

## Funding

This work was supported in part by the National Key Research and Development Program of China (No. 2021YFD2200304-2), the National Natural Science Foundation of China (No. 31470674) and the State Key Laboratory of Subtropical Silviculture (No. KF201902).

## Acknowledgments

We thank Dr. Naresh Vasupalli, State Key Laboratory of Subtropical Silviculture, Zhejiang A & F University, for helping to revise the manuscript.

## Conflict of interest

The authors declare that the research was conducted in the absence of any commercial or financial relationships that could be construed as a potential conflict of interest.

## Publisher’s note

All claims expressed in this article are solely those of the authors and do not necessarily represent those of their affiliated organizations, or those of the publisher, the editors and the reviewers. Any product that may be evaluated in this article, or claim that may be made by its manufacturer, is not guaranteed or endorsed by the publisher.

## Supplementary material

Supplementary Figure 1 OVATE domain alignment of BlOFP proteins.

Supplementary Figure 2 Sequence alignment of AtOFP6, BlOFP3 and BlOFP6.

Supplementary Figure 3 Transgenic *Arabidopsis* plants confirmation with PCR and qRT-PCR. **(A)** PCR amplification of *BlOFP3* and *BlOFP5* genes in transgenic lines and WT. **(B)** PCR amplification of hygromycin and GUS in WT and 35S::BlOFP3 transgenic lines. **(C)** PCR amplification of hygromycin and GUS in WT and 35S::BlOFP5 transgenic lines. **(D)** Expression analysis of *BlOFP3* in 35S::BlOFP3 transgenic lines by qRT-PCR. **(E)** Expression analysis of *BlOFP5* in 35S::BlOFP5 transgenic lines by qRT-PCR. The size of the PCR products indicated in Kb. The asterisk indicates significant difference compared to WT at *p*<0.05 level.

Supplementary Figure 4 Expression analysis of *AtOFP6* in transgenic plants.

## References

[B1] Belles-Boix E. Hamant O. Witiak S. M. Morin H. Traas J. Pautot V. (2006). KNAT6: An arabidopsis homeobox gene involved in meristem activity and organ separation. Plant Cell 18 (8), 1900–1907. doi: 10.1105/tpc.106.041988 16798887 PMC1533965

[B2] Blanco F. Salinas P. Cecchini N. M. Jordana X. Van Hummelen P. Alvarez M. E.. (2009). Early genomic responses to salicylic acid in arabidopsis. Plant Mol. Biol. 70, 79–102. doi: 10.1007/s11103-009-9458-1 19199050

[B3] Cai M. Huang H. Ni F. Tong Z. Lin E. Zhu M. (2018). RNA-Seq analysis of differential gene expression in betula luminifera xylem during the early stages of tension wood formation. PeerJ 6, e5427. doi: 10.7717/peerj.5427 30155351 PMC6108316

[B4] Chen C. Chen H. Zhang Y. Thomas H. R. Frank M. H. He Y.. (2020). TBtools: An integrative toolkit developed for interactive analyses of big biological data. Mol. Plant 13, 1194–1202. doi: 10.1016/j.molp.2020.06.009 32585190

[B5] Clough S. J. Bent A. F. (1998). Floral dip: A simplified method for agrobacterium-mediated transformation of arabidopsis thaliana. Plant J. 16, 735–743. doi: 10.1046/j.1365-313x.1998.00343.x 10069079

[B6] Forslund K. Sonnhammer E.L.J.E.G. (2012). “Evolution of protein domain architectures,” in Evolutionary genomics. methods in molecular biology, vol. 856. Ed. Anisimova M. (Totowa, New Jersey, USA: Humana Press), 187–216. 10.1007/978-1-61779-585-5_8 22399460

[B7] Hackbusch J. Richter K. Müller J. Salamini F. Uhrig J. F. (2005). A central role of arabidopsis thaliana ovate family proteins in networking and subcellular localization of 3-aa loop extension homeodomain proteins. PNAS 102, 4908–4912. doi: 10.1073/pnas.0501181102 15781858 PMC555730

[B8] Hamant O. Pautot V. (2010). Plant development: A TALE story. C. R. Biol. 333 (4), 371–381. doi: 10.1016/j.crvi.2010.01.015 20371112

[B9] Hasson A. Plessis A. Blein T. Adroher B. Grigg S. Tsiantis M.. (2011). Evolution and diverse roles of the CUP-SHAPED COTYLEDON genes in arabidopsis leaf development. Plant Cell 23 (1), 54–68. doi: 10.1105/tpc.110.081448 21258003 PMC3051246

[B10] Hay A. Barkoulas M. Tsiantis M. (2006). ASYMMETRIC LEAVES1 and auxin activities converge to repress *BREVIPEDICELLUS* expression and promote leaf development in *Arabidopsis* . 133, 20, 3955–3961. doi: 10.1242/dev.02545 16971475

[B11] Hay A. Tsiantis M. (2009). A KNOX family tale. Curr. Opin. Plant Biol. 12 (5), 593–598. doi: 10.1016/j.pbi.2009.06.006 19632142

[B12] Hay A. Tsiantis M. (2010). KNOX genes: Versatile regulators of plant development and diversity. Development 137 (19), 3153–3165. doi: 10.1242/dev.030049 20823061

[B13] Knaap E. V. D. Ostergaard L. (2018). Shaping a fruit: Developmental pathways that impact growth patterns. Semin. Cell Dev. Biol. 79, 27–36. doi: 10.1016/j.semcdb.2017.10.028 29092788

[B14] Kumar R. Kushalappa K. Godt D. Pidkowich M. S. Pastorelli S. Hepworth S. R.. (2007). The arabidopsis BEL1-LIKE HOMEODOMAIN proteins SAW1 and SAW2 act redundantly to regulate KNOX expression spatially in leaf margins. Plant Cell 19 (9), 2719–2735. doi: 10.1105/tpc.106.048769 17873098 PMC2048708

[B15] Kumar S. Stecher G. Li M. Knyaz C. Tamura K. (2018). MEGA X: Molecular evolutionary genetics analysis across computing platforms. Mol. Biol. Evol. 35, 1547–1549. doi: 10.1093/molbev/msy096 29722887 PMC5967553

[B16] Li E. Bhargava A. Qiang W. Friedmann M. C. Forneris N. Savidge R. A.. (2012). The class II KNOX gene KNAT7 negatively regulates secondary wall formation in arabidopsis and is functionally conserved in populus. New Phytol. 194, 102–115. doi: 10.1111/j.1469-8137.2011.04016.x 22236040

[B17] Li H. Dong Q. Zhu X. Zhao Q. Ran K. (2019). Genome-wide identification, expression, and interaction analysis for ovate family proteins in peach. Mol. Biol. Rep. 46 (4), 3755–3764. doi: 10.1007/s11033-019-04817-4 31028569

[B18] Li Z. Li B. Liu J. Guo Z. Liu Y. Li Y.. (2016). Transcription factors AS1 and AS2 interact with LHP1 to repress KNOX genes in arabidopsis. J. Integr. Plant Biol. 58 (12), 959–970. doi: 10.1111/jipb.12485 27273574

[B19] Li X. Y. Lin E. P. Huang H. H. Niu M. Y. Tong Z. K. Zhang J. H. (2018). Molecular characterization of SQUAMOSA PROMOTER BINDING PROTEIN-LIKE (SPL) gene family in betula luminifera. Front. Plant Sci. 9, 608. doi: 10.3389/fpls.2018.00608 29780401 PMC5945835

[B20] Li Z. Li B. Shen W. H. Huang H. Dong A. (2017). TCP Transcription factors interact with AS2 in the repression of class-I KNOX genes in arabidopsis thaliana. Plant J. 71 (1), 99–107. doi: 10.1111/j.1365-313X.2012.04973.x 22380849

[B21] Liu Y. Douglas C. J. (2015). A role for OVATE FAMILY PROTEIN1 (OFP1) and OFP4 in a BLH6-KNAT7 multi-protein complex regulating secondary cell wall formation in arabidopsis thaliana. Plant Signal Behav. 10, e1033126. doi: 10.1080/15592324.2015.1033126 26107719 PMC4622736

[B22] Liu D. Sun W. Yuan Y. Zhang N. Hayward A. Liu Y.. (2014). Phylogenetic analyses provide the first insights into the evolution of OVATE family proteins in land plants. Ann. Bot. 113, 1219–1233. doi: 10.1093/aob/mcu061 24812252 PMC4030818

[B23] Liu J. Van Eck J. Cong B. Tanksley S. D. (2002). A new class of regulatory genes underlying the cause of pear-shaped tomato fruit. PNAS 99, 13302–13306. doi: 10.1073/pnas.162485999 12242331 PMC130628

[B24] Livak K. J. Schmittgen T. D. (2001). Analysis of relative gene expression data using real-time quantitative PCR and the 2– ΔΔCT method. Methods 25, 402–408. doi: 10.1006/meth.2001.1262 11846609

[B25] Li E. Wang S. Liu Y. Chen J. G. Douglas C. J. (2011). OVATE FAMILY PROTEIN4 (OFP4) interaction with KNAT7 regulates secondary cell wall formation in arabidopsis thaliana. Plant J. 67(2), 328–341. doi: 10.1111/j.1365-313X.2011.04595.x 21457372

[B26] Long J. A. Moan E. I. Medford J. I. Barton M. K. (1996). A member of the KNOTTED class of homeodomain proteins encoded by the STM gene of arabidopsis. Nature 379 (6560), 66–69. doi: 10.1038/379066a0 8538741

[B27] Mistry J. Chuguransky S. Williams L. Qureshi M. Salazar G. A. Sonnhammer E. L.. (2021). Pfam: The protein families database in 2021. Nucleic Acids Res. 49 (D1), D412–D419. doi: 10.1093/nar/gkaa913 33125078 PMC7779014

[B28] Moore A. D. Björklund Å.K. Ekman D. Bornberg-Bauer E. Elofsson A. (2008). Arrangements in the modular evolution of proteins. Trends Biochem. Sci. 33, 444–451. doi: 10.1016/j.tibs.2008.05.008 18656364

[B29] Nakayama H. Nakayama N. Seiki S. Kojima M. Sakakibara H. Sinha N.. (2014). Regulation of the KNOX-GA gene module induces heterophyllic alteration in north American lake cress. Plant Cell 26 (12), 4733–4748. doi: 10.1105/tpc.114.130229 25516600 PMC4311196

[B30] Pagnussat G. C. Yu H. J. Sundaresan V. (2007). Cell-fate switch of synergid to egg cell in arabidopsis eostre mutant embryo sacs arises from misexpression of the BEL1-like homeodomain gene BLH1. Plant Cell 19, 3578–3592. doi: 10.1105/tpc.107.054890 18055603 PMC2174879

[B31] Rodríguez G. R. Muños S. Anderson C. Sim S. C. Michel A. Causse M.. (2011). Distribution of SUN, OVATE, LC, and FAS in the tomato germplasm and the relationship to fruit shape diversity. Plant Physiol. 156 (1), 275–285. doi: 10.1104/pp.110.167577 21441384 PMC3091046

[B32] Rosin F. M. Hart J. K. Horner H. T. Davies P. J. Hannapel D. J. (2003). Overexpression of a knotted-like homeobox gene of potato alters vegetative development by decreasing gibberellin accumulation. Plant Physiol. 132 (1), 106–117. doi: 10.1104/pp.102.015560 12746517 PMC166957

[B33] Salojarvi J. Smolander O. P. Nieminen K. Rajaraman S. Safronov O. Safdari P.. (2017). Genome sequencing and population genomic analyses provide insights into the adaptive landscape of silver birch. Nat. Genet. 49, 904–912. doi: 10.1038/ng.3862 28481341

[B34] Schmitz A. J. Begcy K. Sarath G. Walia H. (2015). Rice ovate family protein 2 (OFP2) alters hormonal homeostasis and vasculature development. Plant Sci. 241, 177–188. doi: 10.1016/j.plantsci.2015.10.011 26706069

[B35] Sun X. Ma Y. Yang C. Li J. (2020). Rice OVATE family protein 6 regulates leaf angle by modulating secondary cell wall biosynthesis. Plant Mol. Biol. 104, 249–261. doi: 10.1007/s11103-020-01039-2 32715397

[B36] Tang Y. Zhang W. Yin Y. L. Feng P. Li H. L. Chang Y. (2018). Expression of ovate family protein 8 affects epicuticular waxes accumulation in arabidopsis thaliana. Bot. Stud. 59, 12. doi: 10.1186/s40529-018-0228-8 29691677 PMC5915979

[B37] Tsaballa A. Pasentsis K. Darzentas N. Tsaftaris A. S. (2011). Multiple evidence for the role of an ovate-like gene in determining fruit shape in pepper. BMC Plant Biol. 11 (1), 1–16. doi: 10.1186/1471-2229-11-46 21401913 PMC3069956

[B38] Wang S. Chang Y. Ellis B. (2016). Overview of OVATE FAMILY PROTEINS, a novel class of plant-specific growth regulators. Front. Plant Sci. 7, 417. doi: 10.3389/fpls.2016.00417 27065353 PMC4814488

[B39] Wang S. Chang Y. Guo J. Chen J. G. (2007). Arabidopsis ovate family protein 1 is a transcriptional repressor that suppresses cell elongation. Plant J. 50, 858–872. doi: 10.1111/j.1365-313X.2007.03096.x 17461792

[B40] Wang S. Chang Y. Guo J. Zeng Q. Ellis B. E. Chen J. G. (2011). Arabidopsis ovate family proteins, a novel transcriptional repressor family, control multiple aspects of plant growth and development. PloS One 6, e23896. doi: 10.1371/journal.pone.0023896 21886836 PMC3160338

[B41] Wang Y. K. Chang W. C. Liu P. F. Hsiao M. K. Lin C. T. Lin S. M.. (2010). Ovate family protein 1 as a plant Ku70 interacting protein involving in DNA double-strand break repair. Plant Mol. Biol. 74, 453–466. doi: 10.1007/s11103-010-9685-5 20844935

[B42] Wang H. Kong F. Zhou C. (2021). From genes to networks: The genetic control of leaf development. J. Integr. Plant Bio. 63 (7), 1181–1196. doi: 10.1111/jipb.13084 33615731

[B43] Wu S. Zhang B. Keyhaninejad N. Rodriguez G. R. Kim H. J. Chakrabarti M.. (2018). A common genetic mechanism underlies morphological diversity in fruits and other plant organs. Nat. Commun. 9, 4734. doi: 10.1038/s41467-018-07216-8 30413711 PMC6226536

[B44] Yang C. Ma Y. He Y. Tian Z. Li J. (2018). OsOFP19 modulates plant architecture by integrating the cell division pattern and brassinosteroid signaling. Plant J. 93, 489–501. doi: 10.1111/tpj.13793 29205590

[B45] Yang C. Shen W. He Y. Tian Z. Li J. (2016). OVATE family protein 8 positively mediates brassinosteroid signaling through interacting with the GSK3-like kinase in rice. PloS Genet. 12, e1006118. doi: 10.1371/journal.pgen.1006118 27332964 PMC4917237

[B46] Yoo S. D. Cho Y. H. Sheen J. (2007). Arabidopsis mesophyll protoplasts: A versatile cell system for transient gene expression analysis. Nat. Protoc. 2, 1565–1572. doi: 10.1038/nprot.2007.199 17585298

[B47] Zhang L. Zhang X. Ju H. Chen J. Wang S. Wang H.. (2016). Ovate family protein1 interaction with BLH3 regulates transition timing from vegetative to reproductive phase in arabidopsis. Biochem. Biophys. Res. Commun. 470, 492–497. doi: 10.1016/j.bbrc.2016.01.135 26809096

[B48] Zhou S. Cheng X. Li F. Feng P. Hu G. Chen G.. (2019). Overexpression of SlOFP20 in tomato affects plant growth, chlorophyll accumulation, and leaf senescence. Front. Plant Sci. 10, 510. doi: 10.3389/fpls.2019.01510 31850017 PMC6896838

[B49] Zhou R. Fan M. Zhao M. Jiang X. Liu Q. (2022). Overexpression of *LtKNOX1* from *Lilium tsingtauense* in *Nicotiana benthamiana* affects the development of leaf morphology. Plant Signal Behav. 17, 1. doi: 10.1080/15592324.2022.2031783 PMC9176240 35139775

