# Supplementary figures and images for: Genome-wide identification and characterization of OVATE family proteins in Betula luminifera reveals involvement of BlOFP3 and BlOFP5 genes in leaf development
Source: Front Plant Sci. 2022 Oct 13;13:950936. doi: 10.3389/fpls.2022.950936 (PMC9613114; doi:10.3389/fpls.2022.950936)

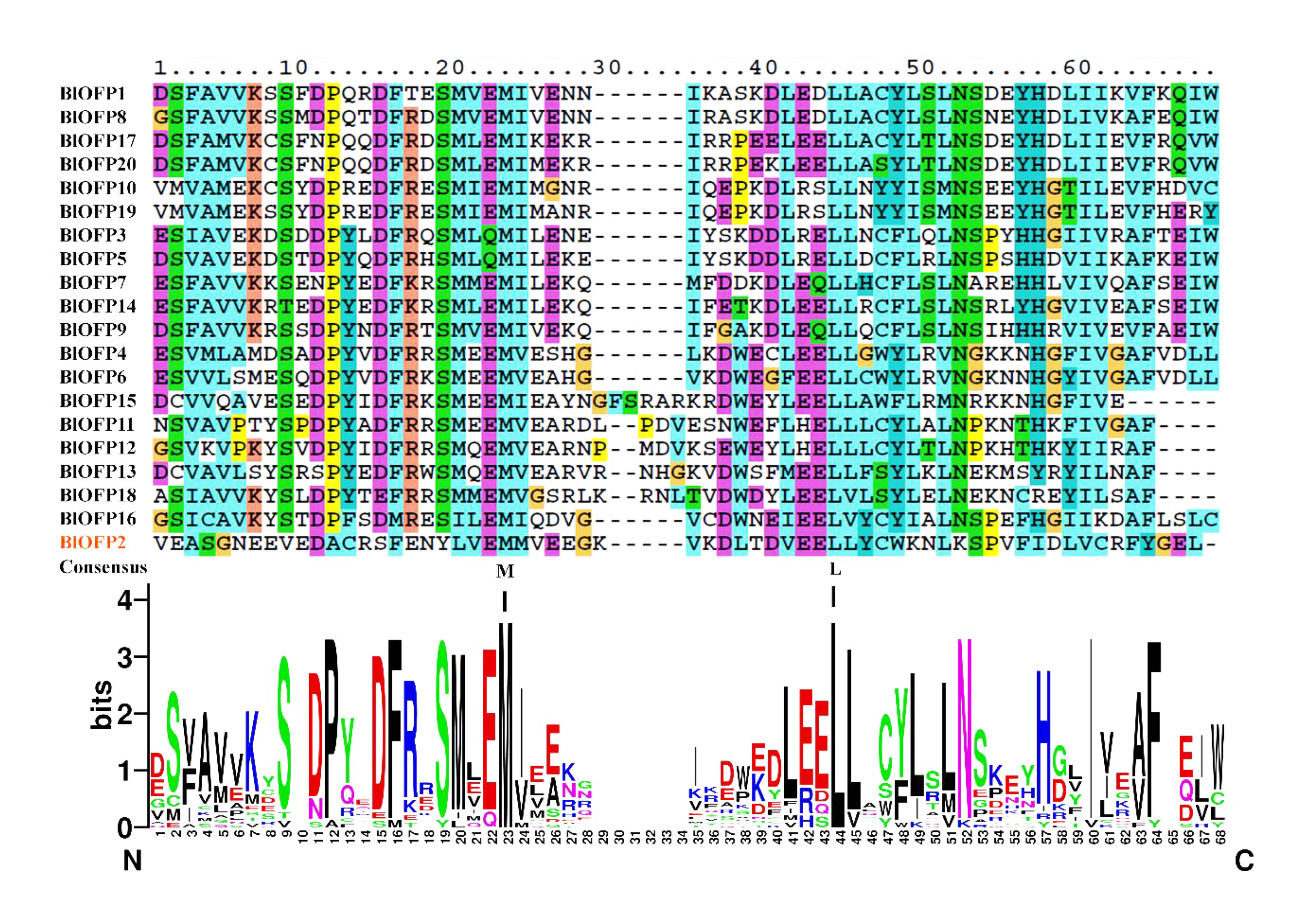

Supplement: Supplementary Figure 1 — OVATE domain alignment of BlOFP proteins. [file Image_1.tif]

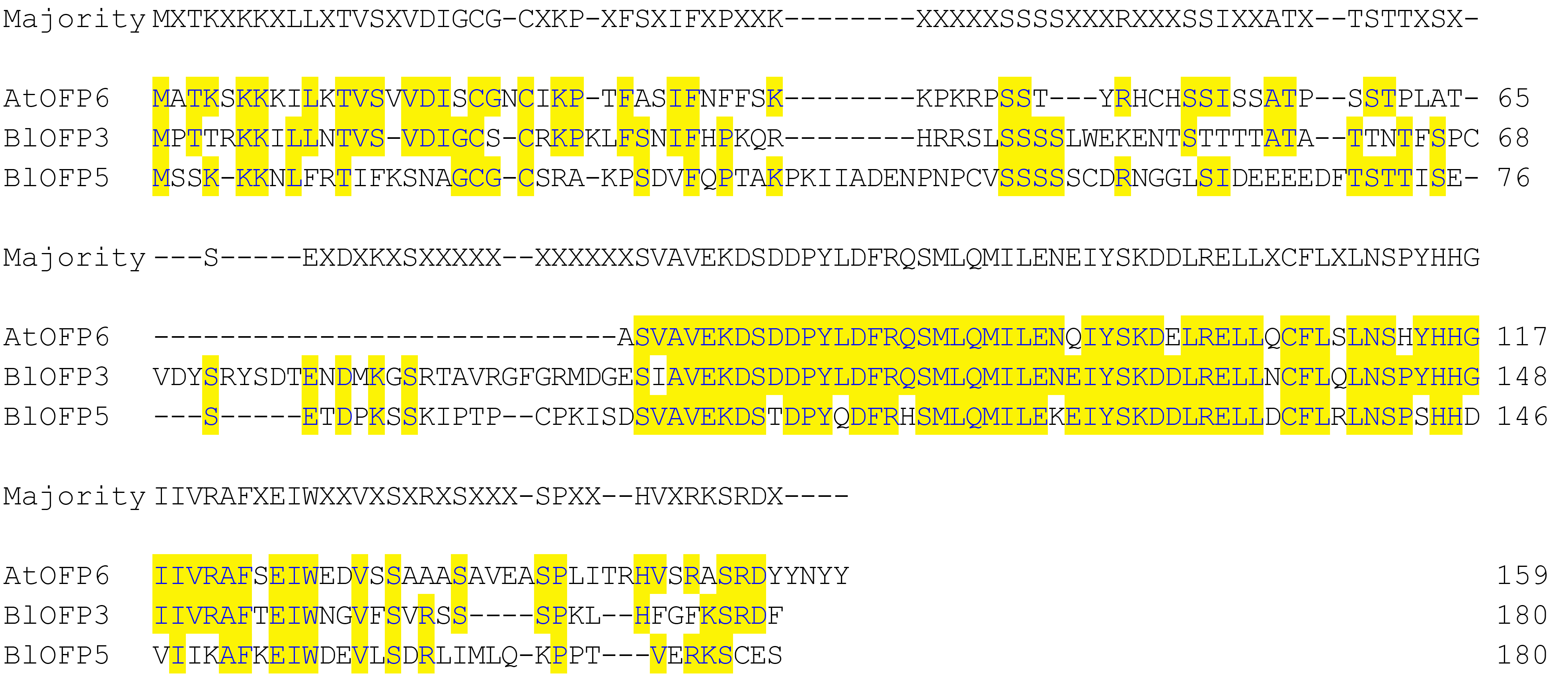

Supplement: Supplementary Figure 2 — Sequence alignment of AtOFP6, BlOFP3 and BlOFP6. [file Image_2.tif]

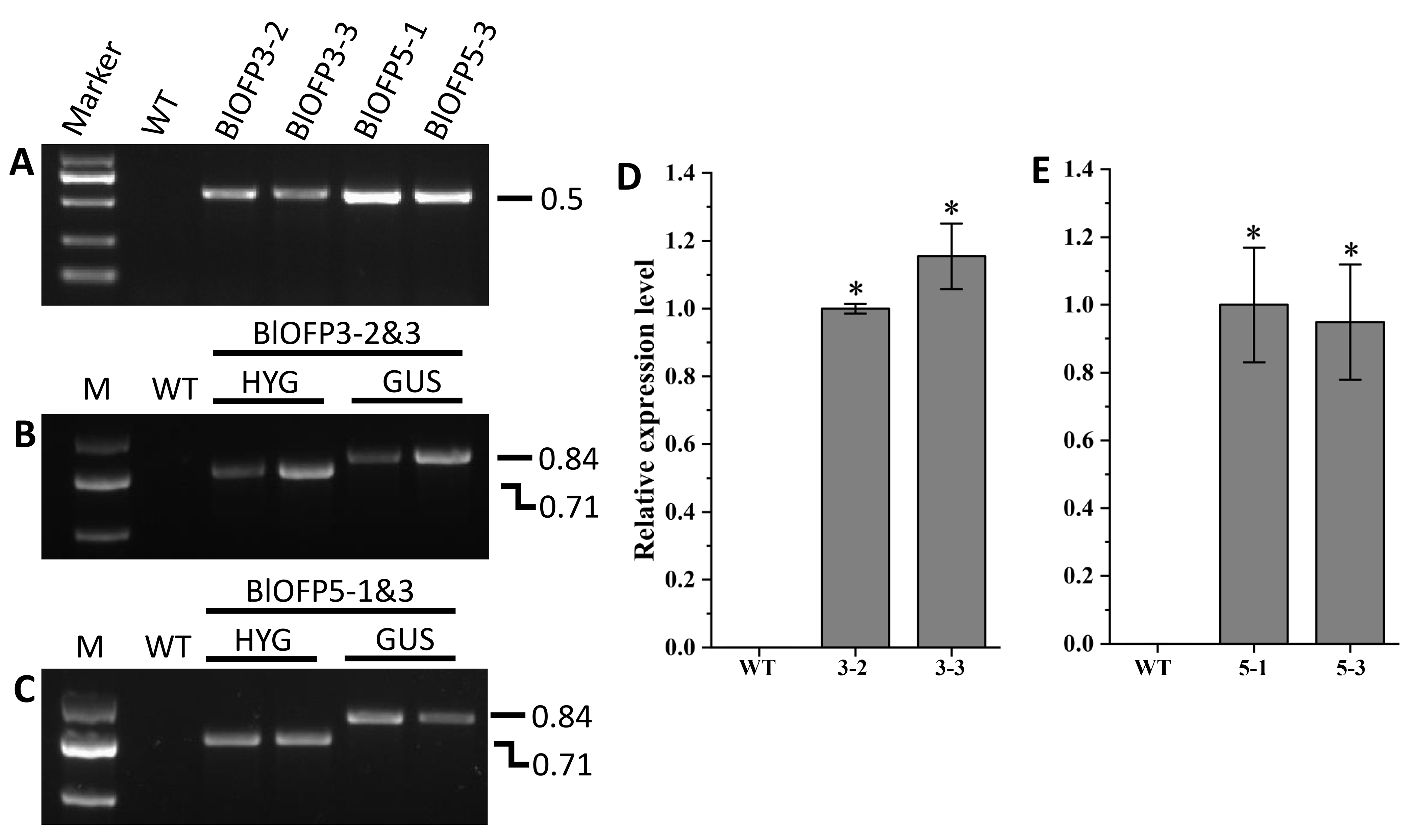

Supplement: Supplementary Figure 3 — Transgenic Arabidopsis plants confirmation with PCR and qRT-PCR. (A) PCR amplification of BlOFP3 and BlOFP5 genes in transgenic lines and WT. (B) PCR amplification of hygromycin and GUS in WT and 35S::BlOFP3 transgenic lines. (C) PCR amplification of hygromycin and GUS in WT and 35S::BlOFP5 transgenic lines. (D) Expression analysis of BlOFP3 in 35S::BlOFP3 transgenic lines by qRT-PCR. (E) Expression analysis of BlOFP5 in 35S::BlOFP5 transgenic lines by qRT-PCR. The size of the PCR products indicated in Kb. The asterisk indicates significant difference compared to WT at p<0.05 level. [file Image_3.tif]

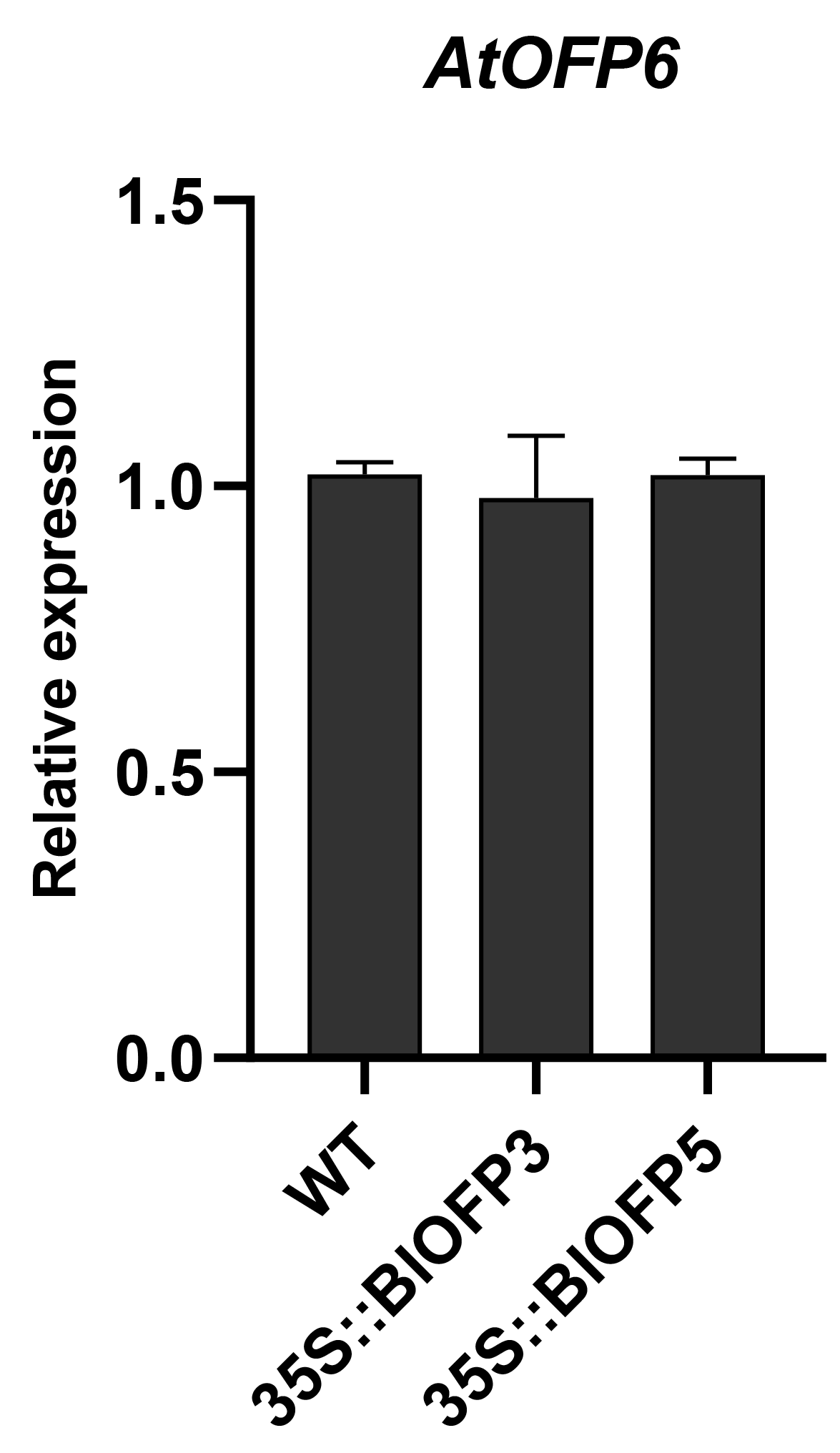

Supplement: Supplementary Figure 4 — Expression analysis of AtOFP6 in transgenic plants. [file Image_4.tif]
